# Appendiceal Neuroendocrine Tumor Masquerading as Acute Appendicitis in a Young Male: A Case Report and Clinical Discussion

**DOI:** 10.2174/0115734056482353260729113227

**Published:** 2026-08-05

**Authors:** Qian Wang, Yanyu Li, Chenxi Wu, Jiang Zhu

**Affiliations:** 1 Department of Ultrasound Medicine, The First People’s Hospital of Linhai City, Linhai, Zhejiang 317000, P.R. China; 2 Department of Pathology Medicine, The First People’s Hospital of Linhai City, Linhai, Zhejiang 317000, P.R. China; 3 Department of Ultrasound, Women's Hospital, Zhejiang University School of Medicine, Hangzhou, China; 4 Zhejiang Provincial Key Laboratory of Precision Diagnosis and Therapy for Major Gynecological Diseases, Women's Hospital, Zhejiang University School of Medicine, Hangzhou, China

**Keywords:** Appendiceal neoplasms, Neuroendocrine tumors, Acute appendicitis, Diagnostic accuracy, Abdominal pain, Inflammatory markers, Diagnosis, Therapy, Prognosis

## Abstract

**Background:**

Appendiceal neuroendocrine neoplasms (ANENs) are rare tumors of the appendix, most of which are low-grade malignancies. They are often discovered incidentally during surgery for acute appendicitis. This report presents a case of a 29-year-old male with typical manifestations of acute appendicitis. Preoperative imaging and laboratory tests were consistent with acute appendicitis with abscess formation, but postoperative pathology revealed an unexpected appendiceal neuroendocrine tumor. The aim of this report is to improve clinicians’ understanding, diagnostic accuracy, and therapeutic decision-making for this condition.

**Case Presentation:**

A 29-year-old male patient presented with a one-day history of migratory right lower quadrant abdominal pain. Physical examination revealed tenderness and rebound tenderness at McBurney’s point, and a positive Rovsing’s sign. Laboratory tests showed elevated inflammatory markers (procalcitonin, interleukin-6, ferritin), consistent with acute appendicitis. Ultrasound and CT imaging revealed a thickened appendix with periappendiceal exudation, consistent with acute appendicitis with abscess formation. Emergency laparoscopic appendectomy was performed. Postoperative pathology confirmed an appendiceal neuroendocrine tumor (G1) with serosal and perineural invasion, without lymphovascular invasion, and with negative margins. The Ki-67 index was 2%+ (G1 according to the 2019 WHO classification). The patient recovered uneventfully, and a 3-month follow-up CT showed no signs of recurrence or metastasis.

**Conclusion:**

This case suggests that simple appendectomy with close surveillance might be considered as an individualized strategy for selected patients with intermediate-risk G1 tumors measuring 1-2 cm with serosal or perineural invasion but without lymphovascular invasion and with negative margins. However, further validation with larger studies and longer follow-up is needed.

## 1 INTRODUCTION

Appendiceal neuroendocrine neoplasms (ANENs) account for approximately 0.3-0.9% of all appendiceal tumors, and the incidence has been increasing in recent years, especially in younger populations [1,2]. Based on histopathological features, neuroendocrine neoplasms of the appendix are classified into classic carcinoid tumors, goblet cell carcinoids (GCCs), and mixed adenoneuroendocrine tumors (MANETs). Classic carcinoid tumors are mostly well-differentiated, slow-growing, and have a good prognosis. In contrast, GCCs and MANETs are more aggressive [3].

The clinical presentation is often non-specific. In more than 80% of cases, the disease is discovered incidentally on pathological examination after appendectomy for acute appendicitis [2]. Imaging plays an important adjunctive role in diagnosis. Ultrasound, computed tomography (CT), and magnetic resonance imaging (MRI) are mainly used to evaluate appendicitis and its complications, but their direct diagnostic value for the tumor itself is limited. The use of right hemicolectomy in patients with tumors measuring 1-2 cm in diameter remains controversial. Treatment decisions should be individualized based on multiple factors, including tumor size, depth of invasion, presence or absence of lymphovascular or perineural invasion, and lymph node metastasis [4].

This article presents a case of ANENs complicated by acute appendicitis and reviews the relevant literature. The clinical manifestations, imaging features, diagnostic challenges, treatment options, and follow-up strategies are systematically described to provide a clinical reference for diagnosis and treatment.

## 2 CASE PRESENTATION

### 2.1 Patient Information

A 29-year-old male patient presented with a one-day history of migratory right lower quadrant abdominal pain. The pain started around the umbilicus and then localized to the right lower quadrant, becoming persistent and intensified, accompanied by nausea and vomiting. Physical examination revealed tenderness and rebound tenderness at McBurney’s point with guarding, and a positive Rovsing’s sign (colon inflation test). Laboratory tests showed elevated inflammatory markers: interleukin-6 274.19 pg/mL (normal range 0.00-7.00), procalcitonin 3.10 ng/mL (normal <0.50), and ferritin 663.0 ng/mL (normal 30.0-400.0), consistent with acute appendicitis.

### 2.2 Preoperative Imaging Studies

Ultrasound examination revealed an enlarged appendix. At its tip, there was a mixed-echo area measuring approximately 23 × 20 mm, with blurred margins and heterogeneous internal echogenicity. Color Doppler Flow Imaging (CDFI) showed abundant peripheral blood flow signals around this area, suggesting acute appendicitis with abscess formation (Fig. **1**).

A non-contrast CT of the abdomen showed a significantly thickened appendix (diameter ~15 mm) with diffuse wall thickening and edema, blurred periappendiceal fat planes, and extensive flocculent exudation, consistent with acute appendicitis with periappendiceal abscess formation (Fig. **2**).

### 2.3 Surgical and Pathological Findings

Based on the clinical manifestations, signs, and imaging findings, the clinical diagnosis was acute gangrenous appendicitis with perforation and acute diffuse peritonitis. Emergency laparoscopic appendectomy was performed. The tumor was not suspected intraoperatively because imaging showed only inflammatory changes of acute appendicitis without any identifiable mass.

Intraoperatively, the appendix was markedly congested and swollen, with gangrenous perforation at its tip. Purulent exudate was observed in the abdominal and pelvic cavities. The appendix measured 4.0 cm in length and 1.0-1.3 cm in diameter. Microscopic examination revealed a 1.0 × 0.8 cm tumor at the tip of the appendix, infiltrating the serosal layer with perineural invasion. No lymphovascular invasion was identified, and the proximal margin was negative (Fig. **3**). Immunohistochemistry showed positivity for CKpan, CDX2, CD56, synaptophysin, and INSM1, with a Ki-67 proliferation index of 2%+ (estimated by counting positive cells per 500 tumor cells in hot spots). According to the 2019 WHO classification, this value falls within the G1 grade (<3%). The pathological diagnosis was appendiceal neuroendocrine neoplasm (grade G1) with acute suppurative appendicitis. Right hemicolectomy was not performed based on the tumor’s pathological characteristics (G1, no lymphovascular invasion, negative margins) and the patient’s preference.

### 2.4 Postoperative Follow-up

The patient recovered uneventfully. At three months postoperatively, contrast-enhanced CT of the abdomen showed minimal exudation and small lymph nodes in the surgical area, which were considered postoperative inflammatory changes. No signs of tumor recurrence or metastasis were observed. The patient is being followed regularly. The key events of the clinical course are outlined in Table **1**.

## 3 DISCUSSION

### 3.1 Disease Overview and Epidemiology

ANENs are the most common malignant tumors of the appendix, accounting for 50-77% of primary appendiceal neoplasms [5]. Their incidence ranges from 0.3 to 0.9% [1] and has been increasing in recent years, possibly due to the widespread use of imaging and heightened awareness of pathological diagnosis [2]. The majority of ANENs are well-differentiated neuroendocrine tumors (NETs, grades G1/G2). The classic carcinoid tumor (grade G1) is the most common subtype, accounting for more than 60% of cases. It is characterized by uniform cell morphology, a low proliferation index (Ki-67 usually <3%), indolent growth, and a favorable prognosis [3].

### 3.2 Clinical Presentation

The clinical presentation of ANENs is highly non-specific and often overlaps with common inflammatory conditions. The majority of patients (>80%) have no specific symptoms, and tumors are often incidentally found on pathological examination after emergency surgery for acute appendicitis [2]. The present case is typical: a young man presented with classic migratory right lower quadrant pain; physical signs and laboratory findings strongly supported acute appendicitis, and no tumor was suspected preoperatively. It has been reported that up to 91.4% of patients with appendiceal malignancies present with symptoms resembling acute appendicitis, resulting in a high rate of clinical misdiagnosis [6]. Carcinoid syndrome from liver metastases or obstructive symptoms from large tumors occurs only in a minority of advanced cases; these scenarios are rare.

### 3.3 Imaging Characteristics

Imaging has limited value in the preoperative diagnosis of ANENs and is used primarily to assess the severity and complications of appendicitis [7].

Ultrasound Examination: Ultrasound often demonstrates appendiceal enlargement, wall thickening, and surrounding fluid or abscess. It may occasionally reveal spherical enlargement of the appendiceal tip or focal thickening of the body in classic carcinoid tumors. However, in most cases, the echogenic features are indistinguishable from those of uncomplicated appendicitis or abscess [8].

CT Examination: CT is a key modality for evaluating the acute abdomen. It can clearly demonstrate appendiceal enlargement, fecaliths, periappendiceal fat stranding, and lymphadenopathy. However, ANENs typically lack specific CT features, appearing only as focal wall thickening or mildly enhancing nodules, which are often masked by the surrounding acute inflammation [9]. Therefore, the preoperative diagnostic yield of imaging for ANENs remains limited, and definitive diagnosis relies on postoperative histopathology.

### 3.4 Role of Intraoperative Frozen Section (IFS)

Most ANENs are discovered incidentally during surgery for acute appendicitis [2]. Intraoperative Frozen Section (IFS) is not part of the standard workup for suspected appendicitis. However, when a solid mass or focal thickening is visible at the appendiceal base or tip, IFS can be valuable. It allows immediate assessment of tumor size and margin status. For tumors clearly >2 cm or with gross base involvement, a positive IFS finding can guide the surgeon to perform an immediate right hemicolectomy, avoiding a second operation. Nevertheless, IFS has important limitations: it cannot accurately assess the Ki-67 index or subtle perineural/lymphovascular invasion, which are critical for risk stratification of 1-2 cm intermediate-risk tumors such as the one presented in this report. Therefore, while IFS may support intraoperative decisions, definitive grading and treatment planning must rely on permanent pathology and immunohistochemistry [4]. In our case, because no macroscopic tumor was suspected intraoperatively, IFS was not performed, which is consistent with standard clinical practice.

### 3.5 Diagnostic Challenges and Differential Diagnosis

Preoperative diagnosis of ANENs is challenging because the symptoms, imaging findings, and routine laboratory tests lack specificity and may mimic other common right lower quadrant emergencies. Definitive diagnosis requires postoperative histopathology. The differential diagnosis should focus on the following entities:

#### 3.5.1 Appendiceal Mucinous Adenocarcinoma

Imaging often shows a cystic mass in the right lower quadrant with thick, irregular walls and internal septations. A characteristic finding is pseudomyxoma peritonei resulting from mucin penetrating the serosa, manifesting as diffuse mucinous masses or ascites. This differs from the more localized solid or inflammatory mass typical of ANENs [10]. Laboratory tests may show elevated carcinoembryonic antigen (CEA) and CA19-9.

#### 3.5.2 Appendiceal Adenocarcinoma

This tumor frequently arises at the base of the appendix. Imaging shows irregular thickening or a mass at the base, with a tendency to invade the ileocecal region and colon [11]. Tumor markers such as CEA and CA125 usually have higher positivity rates compared to ANENs.

#### 3.5.3 Metastatic Neuroendocrine Tumors

When diagnosing ANENs, it is important to exclude the possibility of metastasis from a neuroendocrine primary elsewhere, such as the small intestine or pancreas. Differentiation requires comprehensive imaging to identify the primary tumor, and immunohistochemistry (*e.g*., CDX2, SATB2) provides valuable ancillary information.

### 3.6 Pathological Grading and Classification

According to the 2019 WHO classification, gastrointestinal NETs are graded based on the Ki-67 proliferation index into G1 (<3%), G2 (3-20%), and G3 (>20%) [12]. In the present case, the Ki-67 index was 2%+, which falls within the G1 grade (<3%). Immunohistochemical positivity for synaptophysin and CDX2 supported the diagnosis of neuroendocrine differentiation.

### 3.7 Treatment Strategies and Controversies

The management of ANENs should be stratified based on tumor size and the presence of high-risk pathological factors.

Key risk factors for considering right hemicolectomy include: tumor size >2 cm, G2/G3 grade, lymphovascular invasion, mesoappendiceal invasion >3 mm, positive margins, and tumor location at the base.

#### 3.7.1 Tumors <1 cm (Low-risk)

Simple appendectomy with R0 resection is usually curative, and additional surgery or intensive follow-up is generally not needed [13].

#### 3.7.2 Tumors >2 cm or with High-risk Features

(*e.g*., lymphovascular invasion, G2/G3 grade, positive margins, mesoappendiceal invasion >3 mm): Right hemicolectomy with regional lymphadenectomy is recommended [14].

#### 3.7.3 Tumors 1-2 cm (Intermediate-risk)

This remains an area of controversy. Traditional guidelines suggest considering right hemicolectomy when high-risk factors are present. However, recent studies indicate that for a G1 tumor measuring 1-2 cm with only serosal or perineural invasion but without lymphovascular invasion and with negative margins, simple appendectomy may be sufficient. Adding right hemicolectomy does not significantly improve overall survival and may increase the risk of complications [15-17].

Serosal or perineural invasion alone, in the absence of lymphovascular invasion or other high-risk features, remains a controversial factor and is not considered an absolute indication for right hemicolectomy.

In this case, the tumor measured 1.0 cm. Although it infiltrated the serosal layer with perineural invasion, there was no lymphovascular invasion, and the margins were negative. After discussion with the patient, right hemicolectomy was not performed. The absence of lymphovascular invasion was a decisive factor in choosing a conservative surgical approach. Guideline differences exist among academic organizations. The NCCN guidelines recommend simple appendectomy for ANENs <2 cm [18]. In contrast, the ENETS and NANETS guidelines emphasize risk stratification for 1-2 cm tumors: appendectomy for low-risk patients and right hemicolectomy for high-risk patients [19,20]. These differences highlight the need for more prospective studies to establish consensus.

### 3.8 Prognosis and Follow-up

Localized ANENs have an excellent prognosis, with a 5-year survival rate exceeding 95% [21]. Approximately 5-10% of cases may experience recurrence or metastasis, often associated with initial high-risk pathological features. Follow-up should be based on risk stratification:

Low-risk patients (tumor <1 cm and no high-risk factors) do not require routine follow-up.

For intermediate-risk patients (such as the present case), annual abdominal imaging (CT or MRI) for 5-10 years is recommended [22].

Biochemical markers such as chromogranin A (CgA) or urinary 5-hydroxyindoleacetic acid (5-HIAA) have low sensitivity in localized disease and are not recommended for routine monitoring [23].

## 4 STUDY LIMITATIONS

Several limitations should be acknowledged. First, as a single case report, the findings may not be generalizable to all patients with ANENs. Second, the follow-up period of three months is relatively short for a low-grade malignancy with indolent growth; long-term surveillance (*e.g*., 5-10 years) is necessary to confirm the absence of late recurrence. Third, the decision to forgo right hemicolectomy was based on tumor characteristics (G1, negative margins, no lymphovascular invasion) and the patient’s preference; current guidelines remain controversial for 1-2 cm tumors, and more prospective studies are needed to validate this individualized approach.

## CONCLUSION

Appendiceal neuroendocrine tumors are often concealed within acute appendicitis, and the diagnosis is frequently an incidental postoperative finding. This case suggests that simple appendectomy with close surveillance might be considered as an individualized strategy for selected patients with intermediate-risk G1 tumors measuring 1-2 cm with serosal or perineural invasion but without lymphovascular invasion and with negative margins. Standardized postoperative pathological examination and risk-stratified long-term follow-up are essential to improving patient prognosis. However, further validation with larger studies and longer follow-up is needed.

## Figures and Tables

**Fig. (1):**
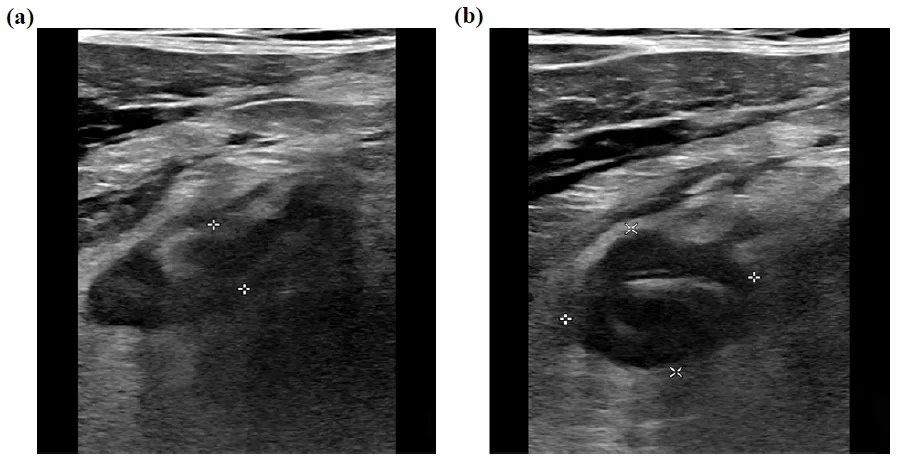
Transabdominal ultrasound images. **(a)** Long-axis view shows a thickened appendix. **(b)** Short-axis view reveals a mixed-echoic abscess at its tip. These findings were consistent with acute appendicitis but did not suggest an underlying tumor.

**Fig. (2):**
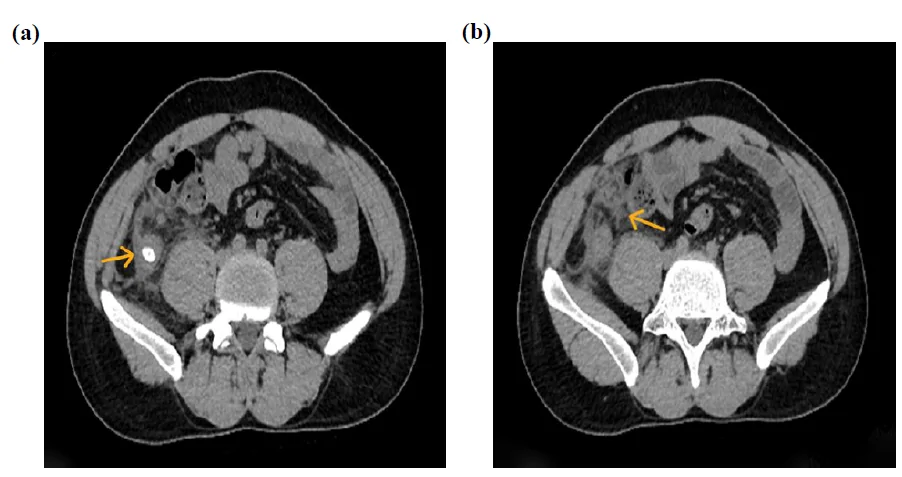
Axial non-contrast CT images of the abdomen. **(a)** A thickened appendiceal wall with an intraluminal fecalith (yellow arrow). **(b)** Periappendiceal fat stranding and exudation (yellow arrow). No mass was evident preoperatively.

**Fig. (3):**
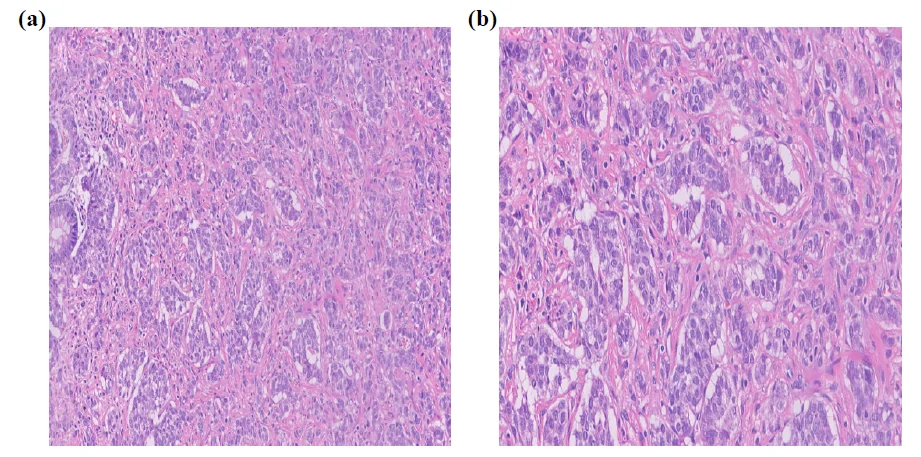
Histopathology of the appendiceal tumor.**(a, b)** Hematoxylin and eosin (H&E) staining demonstrates a well-differentiated neuroendocrine tumor with a nested growth pattern, invading the serosa and perineural spaces (original magnification: **(a)** ×10; **(b)** ×20).

**Table 1:** Timeline of clinical course.

**Time Point**	**Event**
Day 1	Admission with acute right lower quadrant abdominal pain. Ultrasound and CT performed; acute appendicitis with an abscess diagnosed. Emergency laparoscopic appendectomy.
Day 3	Uneventful discharge
Day 7	Pathology report: ANEN G1
Month 3	Follow-up CT: no recurrence or metastasis; patient continues regular surveillance

## Data Availability

The data and supportive information are available within the article.

## LIST OF ABBREVIATIONS

ANENs: Appendiceal Neuroendocrine Neoplasms
NETs: Neuroendocrine Tumors
GCCs: Goblet Cell Carcinoids
MANETs: Mixed Adenoneuroendocrine Tumors
CT: Computed Tomography
MRI: Magnetic Resonance Imaging
CDFI: Color Doppler Flow Imaging
CEA: Carcinoembryonic Antigen
IFS: Intraoperative Frozen Section
H&E: Hematoxylin and Eosin

## STANDARDS OF REPORTING

CARE guidelines have been followed.
